# A Synthetic Model of Human Beta-Thalassemia Erythropoiesis Using CD34+ Cells from Healthy Adult Donors

**DOI:** 10.1371/journal.pone.0068307

**Published:** 2013-07-08

**Authors:** Y. Terry Lee, Ki Soon Kim, Colleen Byrnes, Jaira F. de Vasconcellos, Seung-Jae Noh, Antoinette Rabel, Emily R. Meier, Jeffery L. Miller

**Affiliations:** Molecular Genomics and Therapeutics Section, Molecular Medicine Branch, National Institute of Diabetes and Digestive and Kidney Diseases, National Institutes of Health, Bethesda, Maryland, United States of America; Southern Illinois University School of Medicine, United States of America

## Abstract

Based upon the lack of clinical samples available for research in many laboratories worldwide, a significant gap exists between basic and clinical studies of beta-thalassemia major. To bridge this gap, we developed an artificially engineered model for human beta thalassemia by knocking down beta-globin gene and protein expression in cultured CD34+ cells obtained from healthy adults. Lentiviral-mediated transduction of beta-globin shRNA (beta-KD) caused imbalanced globin chain production. Beta-globin mRNA was reduced by 90% compared to controls, while alpha-globin mRNA levels were maintained. HPLC analyses revealed a 96% reduction in HbA with only a minor increase in HbF. During the terminal phases of differentiation (culture days 14–21), beta-KD cells demonstrated increased levels of insoluble alpha-globin, as well as activated caspase-3. The majority of the beta-KD cells underwent apoptosis around the polychromatophilic stage of maturation. GDF15, a marker of ineffective erythropoiesis in humans with thalassemia, was significantly increased in the culture supernatants from the beta-KD cells. Knockdown of beta-globin expression in cultured primary human erythroblasts provides a robust *ex vivo* model for beta-thalassemia.

## Introduction

Inherited mutations or deletions at the HBB gene locus on chromosome 11p15.4 cause beta-thalassemia [1]. The heterozygous state is prevalent in tropical regions and likely plays a role in protecting carrier populations from malarial disease [2]. There are over 200 mutations or deletions that cause a beta-thalassemia phenotype [3]. Homozygous or compound heterozygous inheritance causes more severe reductions in beta-globin gene and protein expression. When hemoglobin production becomes insufficient for the delivery of oxygen, regular and lifelong erythrocyte transfusions are required.

Microcytic hypochromic anemia usually develops in beta-thalassemia during infancy or early childhood with the developmental loss of fetal hemoglobin expression. The pathophysiology of this anemia is multifactorial and includes shortened survival of erythrocytes in the peripheral blood. In addition, so-called “ineffective” erythropoiesis develops despite increased erythropoietin levels and packing of the marrow with erythroblasts. In human beta-thalassemia, apoptotic cell death [4] is a major mechanism by which there is a decrease in the number of generated erythroblast precursor cells [5].

Studies of the molecular and cellular features of the beta-thalassemia phenotype are aimed toward novel diagnostic and therapeutic strategies. Efforts have been made to better understand the causes of ineffective erythropoiesis with clinical goals of improving red cell production and reducing the burden of iron overload. Our understanding of this cellular process remains vague, in part, because bone marrow sampling is not regularly performed. Major differences in the clinical phenotype also exist between thalassemic patients with identical globin mutations. As an alternative for *in vivo* studies, murine models of thalassemia were developed, but differences between the murine models and human beta-thalassemia major were reported [6]–[8]. In this study, a synthetic model for human beta-thalassemia erythropoiesis is reported as an experimental bridge between the non-human models and the clinic. Lentiviral-mediated knockdown of beta-globin expression was studied using CD34+ cells obtained from healthy human adults. This model reiterates several features of ineffective erythropoiesis in human beta-thalassemia including accumulation of insoluble alpha-globin protein and apoptosis at the polychromatophilic stage of differentiation.

## Materials and Methods

### Ethics Statement

Approval for the research protocol and consent documents pertaining to all studies using primary erythroblasts was granted by the Intramural National Institute of Diabetes and Digestive and Kidney Diseases Institutional Review Board. Written informed consent was obtained from all research subjects prior to participation in this study.

### Cell Culture

Cryopreserved CD34+ cells obtained from healthy adult human donors were used for all studies [9]. A 21 day *ex vivo* serum free culture system was utilized that consists of two phases. In culture phase I (culture days 1–7), CD34+ cells were placed in media containing StemPro-34 complete media (l-glutamine, pen-strep and StemPro-34 nutrient supplement) (Invitrogen, Carlsbad, CA) supplemented with 50 ng/ml SCF (HumanZyme, Chicago, IL), 50 ng/ml FLT3-Ligand (HumanZyme) and 10 ng/ml IL-3 (HumanZyme). After 7 days, the cells were transferred to erythropoietin (EPO; Amgen) supplemented medium (phase 2; culture days 7–21) which is comprised of the following: StemPro-34 complete medium, 4 U/ml EPO, 3 µM mifepristone (Sigma Aldrich, St. Louis, MO), 10 µg/ml insulin (Sigma Aldrich), 3 U/ml heparin (Hospira, Inc, Lake Forest, IL) and 0.8 mg/ml holo transferrin (Sigma Aldrich). Purecell NEO Neonatal High Efficiency Leukocyte Reduction Filter for Red Blood Cells (Pall Life Sciences, Ann Arbor, MI) was used to purify enucleated red blood cells on culture day 21.

### Flow Cytometry Analysis

Immunostaining with antibodies directed against CD71 (Invitrogen) and glycophorin A (GPA) (Invitrogen) were performed to assay cell differentiation on culture days 14, 18, and 21 using the BD FACSAria I flow cytometer (BD Biosciences, San Jose, CA) [10]. Positively stained populations of cells were defined by fluorescence of more than two standard deviations above the unstained cells. A minimum of 5000 live cell events was recorded. Apoptosis was analyzed by flow cytometry using the Annexin V: PE Apoptosis Detection Kit I (BD Biosciences) according to manufacturer’s instructions. Intracellular staining was utilized for the detection of the active form of caspase-3, where approximately 500,000 cells were fixed and permeabilized using the BD Cytofix/Cytoperm Fixation/Permeabilization kit (BD Biosciences) and stained with caspase-3 (BD Biosciences), according to the manufacturer’s protocol. Apoptosis was induced in Jurkat cells following treatment with camptothecin (Sigma Aldrich) for use as a positive control.

### Lentiviral shRNA Transduction

Clone TRCN0000232626 5′-CCGGCTTGGACCCAGAGGTTCTTTGCTCGAGCAAAGAACCTCTGGGTCCAAGTTTTTG-3′ (Sigma Aldrich) targeting human beta-globin mRNA was used. Non-targeting shRNA control SHC002V (Sigma Aldrich) served as donor-matched controls. Cryopreserved CD34+ cells were washed and placed in phase I culture medium at an initial concentration of 250,000 cells/ml. After three days, 300,000 cells were transduced in 300 µl of phase I culture medium containing the viral particles (multiplicity of infection of 12). After 24 hours, the cells were resuspended in 4.0 ml phase I culture medium containing 0.5 µg/ml puromycin for an additional three days prior to pelleting and resuspension in 15 ml of phase II culture medium. The phase II culture medium was not supplemented with puromycin, since puromycin selection of mock-transduced cells resulted in complete cell death under these conditions.

### Quantitative PCR Analyses for Gene Expression

On culture day 14, total RNA from three separate donors was extracted using the RNeasy Plus kit (Qiagen, Valentia, CA) and reverse transcribed using SuperScript III reverse transcriptase with Oligo-dT(20) and RNase H treatment (Invitrogen). Real-time PCR was performed on the ABI PRISM 7700 sequence detection system instrument and software (Applied Biosystems, Foster City, CA), using the manufacturer’s recommended conditions. QPCR assays and PCR conditions were performed with primers, probes, or Assays-on-Demand Gene Expression Products (Applied Biosystems) described previously [11]. Individual globin copy numbers were calculated by comparison with standard curves generated from a plasmid DNA encoding each globin template.

### HPLC Analysis of Fetal and Adult Hemoglobin

Cells (1.5×10^6^) were pelleted and resuspended in 120 µl of distilled water followed by lysing through repeated freeze-thaw cycles in a dry ice ethanol bath. Cell debris was removed by centrifugation and filtration through Ultrafree-MC devices (Millipore, Billerica, MA). Samples (80 µl) were analyzed for HbF and HbA using a 204 mm POLYCATA column (Poly LC, Columbia, MD) fitted to a Gilson HPLC system (Gilson, Middleton, WI) [9], [12]. HPLC globin peaks were quantitated and compared using Gilson Unipoint LC software (version 5.11). Total areas under the HbA and HbF peaks were used for ratio comparisons.

### Protein Isolation and Western Analyses

For protein isolation, three million cells were lysed with 250 µl RIPA buffer in the presence of HALT protease inhibitor cocktails (Pierce) according to the manufacturer’s instructions. After centrifugation at 16,000 g for 10 minutes, the soluble and insoluble fractions were collected, and the soluble protein concentrations were measured using Coomassie Plus (Bradford) Assay kit (Pierce). The insoluble fractions were washed with ice cold 1X PBS twice, and solubilized by boiling in 35 µl 2X LDS buffer (Invitrogen) [13].

Equal amounts of protein (20–30 µg) were separated by gel electrophoresis using NuPAGE Novex 4–12*%* Bis-Tris gel in MOPS buffer and transferred using the iBlot Blotting System with nitrocellulose membranes (Invitrogen). Blots were probed with antibodies against human alpha-globin (Santa Cruz Biotechnology, Santa Cruz, CA), beta-globin (Abnova, Walnut, CA), gamma-globin (Abnova), and the appropriate horseradish peroxidase-conjugated secondary antibodies (Santa Cruz Biotechnology). Immunoreactive proteins were detected and visualized using ECL Plus Western detection reagents (GE Healthcare, Pascataway, NJ). Soluble fractions were probed for beta-actin (Abcam, Cambridge, MA) antibody, and insoluble fractions were probed for glycophorin A (Santa Cruz biotechnology) as loading controls. Band intensities were analyzed using the Image J software program (http://rsbweb.nih.gov/ij/).

### ELISA Analysis

Quantification of GDF15 was performed on serum from culture day 21 cells using the DuoSet ELISA for human GDF15 (R&D Systems) following the manufacturer’s protocol [14]. The optical density was read utilizing the ELx808 Absorbance Microplate Reader (BioTek, Winooski, VT).

### Statistical Analysis

Replicate data are expressed as means ± standard deviation (SD) with significance calculated by Student’s *t* test.

## Results

### Globin mRNA Expression Pattern

**Figure 1:**
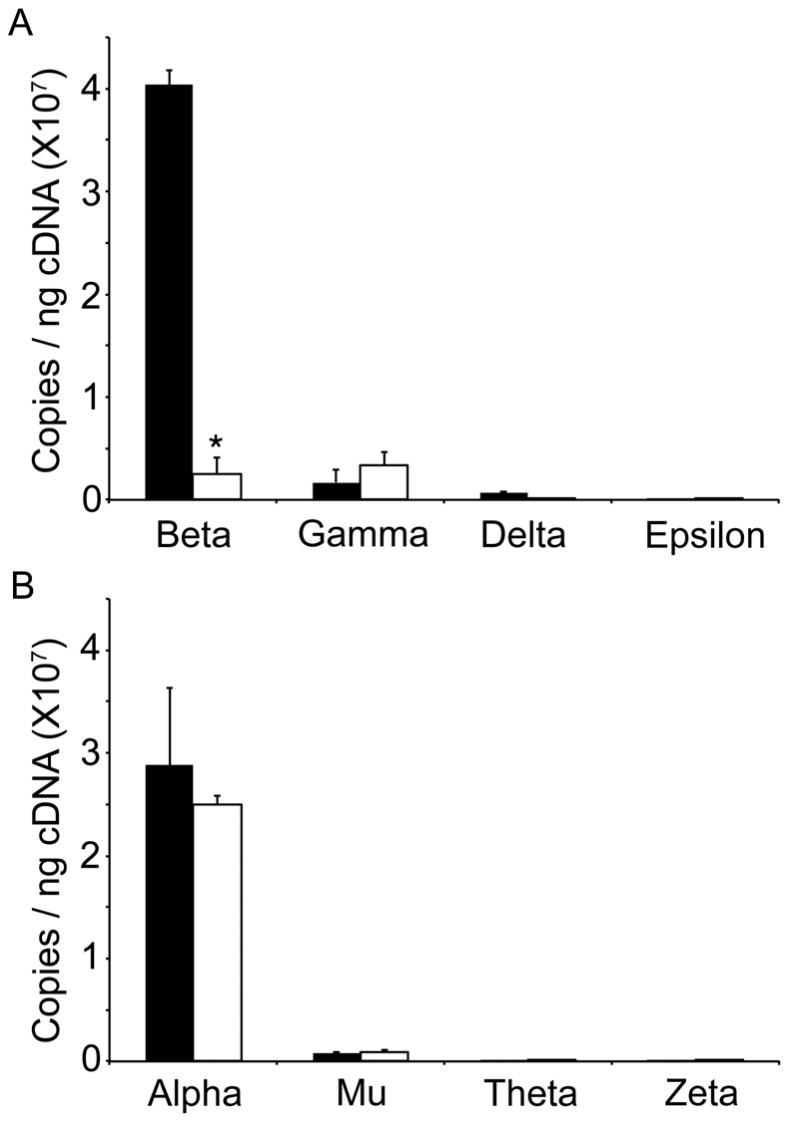
QPCR Quantitation of globin mRNA. RNA samples from erythroblasts cultured on day 14 were examined for globin mRNA expression using quantitative PCR. (A) Expression levels of beta-, gamma-, delta-, and epsilon-globins. (B) Expression levels of alpha-, mu-, theta-, and zeta-globins. Average copy number per ng cDNA is shown on the y-axis from three separate donors, control (black bar) and beta-KD (open bar). Standard deviation bars are shown in vertical lines. Asterisks signify statistical significance of *p*<0.05.

Human CD34+ cells from healthy volunteers were cultured *ex vivo* for 21 days in a two-phase, serum free system to engineer a beta-thalassemia major model. shRNA technology was employed to target silencing of human beta-globin mRNA expression. Informatics analyses of the TRCN0000232626 clone sequence revealed reduced levels of both beta- and delta-globin mRNA. Globin mRNA expression profiles were measured in three separate donors on culture day 14 by QPCR as previously described [11]. Figure 1A shows that greater than 90% of beta-globin mRNA was silenced when compared to the control (non-targeting shRNA, SHC002V) (control = 4.0×10^7^±1.4×10^6^ copies/ng cDNA vs. beta-KD = 2.5×10^6^±1.6×10^6^ copies/ng cDNA, *p* = 0.01). The gamma-globin mRNA demonstrated a less than 2 fold increase in beta-KD when compared to control (control = 1.7×10^6^±1.2×10^6^ copies/ng cDNA vs. beta-KD = 3.4×10^6^±1.4×10^6^ copies/ng cDNA). Delta-globin mRNA showed a 4.5 fold decrease in beta-KD compared to the control (control = 6.9×10^5^±8.3×10^4^ copies/ng cDNA vs. beta-KD = 1.5×10^5^±2.3×10^4^ copies/ng cDNA). There was an insignificant increase in the expression of epsilon-globin mRNA. Alpha-globin locus mRNA expression (alpha-, mu-, theta-, zeta-, globin) shown in Figure 1B demonstrated no significant changes in mRNA levels compared to controls.

### Analysis of Hemoglobin and Cellular Phenotype upon Completion of Cultured Differentiation

**Figure 2:**
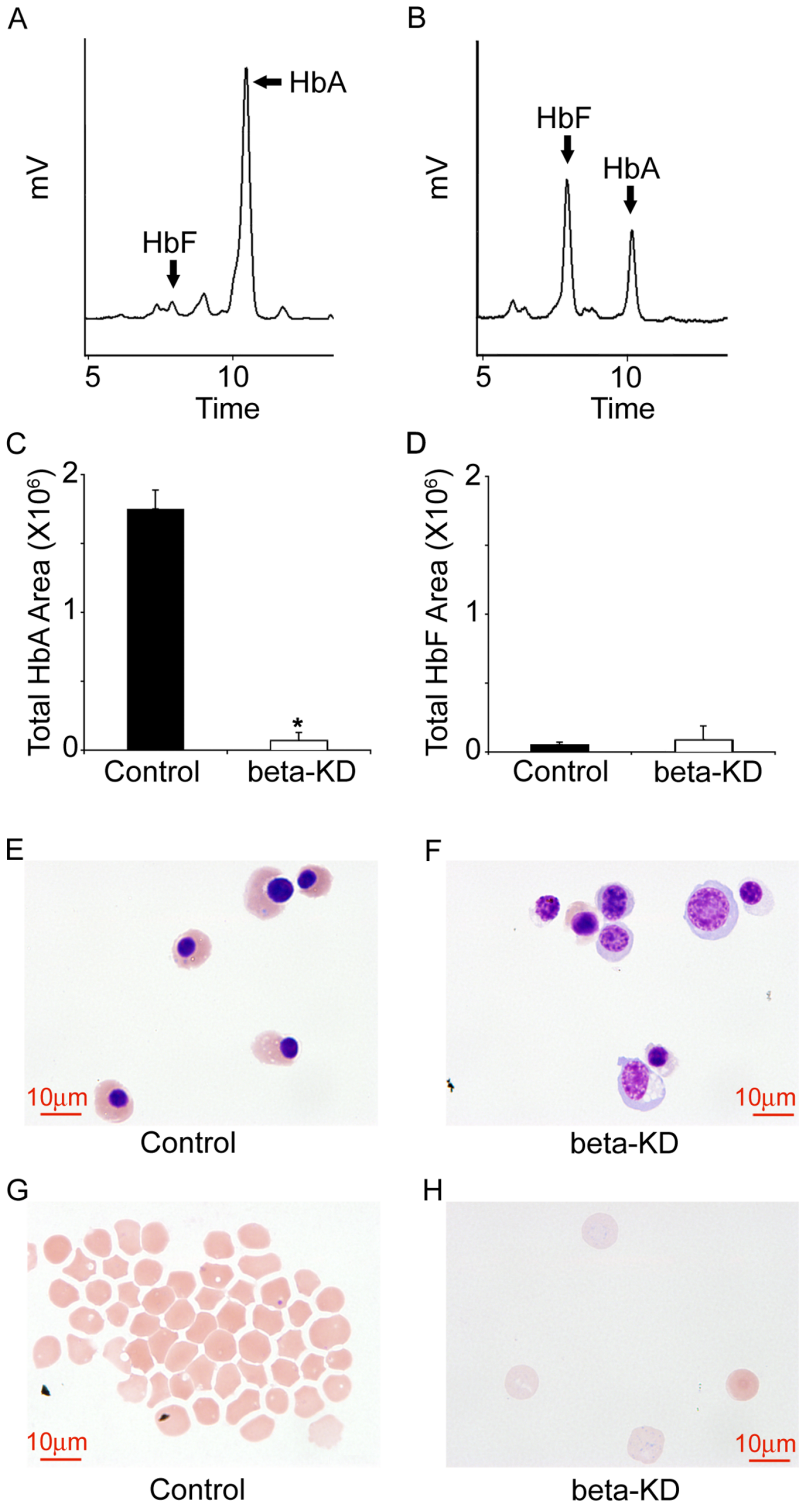
Hemoglobin and globin chain analyses. High performance liquid chromatography analyses of adult hemoglobin (HbA) and fetal hemoglobin (HbF) from culture day 21 erythroblasts (A) Control, (B) beta-KD. Total area under the (C) adult hemoglobin (HbA), and (D) fetal hemoglobin (HbF) peaks was measured using 1.5×10^6^ cultured cells from three donors. Each panel shows average values with standard deviation bars from control (black bar) and beta-KD (open bar). Cytospin preparations of the live cells were stained with Wright-Giemsa on culture day 21 for (E) control cells, (F) beta-KD cells and (G) enucleated control cells, (H) enucleated beta-KD cells with 10µm scale bars. Asterisks signify statistical significance of *p*<0.05.

HPLC was performed from 1.5×10^6^ cells collected from control and beta-KD cells on culture day 21 for measurement of adult (HbA) and fetal hemoglobin (HbF). Representative HPLC tracings are shown from control and beta-KD cells in Figure 2A and B, respectively. As expected, the control cells contained relatively low HbF (2.9±0.7%) levels, but the percentage significantly increased to 49.3±9.3% in the beta-KD cells. Since the increase in the HbF percentage was not reflected in the gamma-globin mRNA, total area under the HbA and HbF peaks were measured in Figure 2C–D. Consistent with the beta-KD reduction in >90% of beta-globin mRNA, the total area measured under the HbA peak was also reduced 11.8 fold (*p*<0.01), and the total area measured under the HbF peak remained relatively unchanged with a slight increase that did not reach statistical significance. Therefore, the increased percentage of HbF shown in Figure 2A–B reflects a significant decrease in the HbA production in the beta-KD cells.

Wright-Giemsa staining of culture day 21 cytospins from control (Figure 2E) showed a main population of orthrochromatic normoblasts. In contrast, the beta-KD cells (Figure 2F) demonstrated a less mature phenotype (polychromatophilic normoblasts). Many abnormal orthrochromatic normoblasts were seen with reduced cytoplasmic volume and decreased hemoglobinization compared to the matched controls. The cellular morphology suggested major erythroid defects around the polychromatophilic-orthochromatic stage of differentiation similar to that identified in human beta-thalassemia marrow [5]. In addition to nucleated cells, the two-phase serum free culture model permitted differentiation into enucleated erythrocytes. Mature erythrocytes were examined after filtering the culture day 21 cells through a leukocyte reduction filter from control and beta-KD cells followed by Wright-Giemsa staining. Figure 2G shows the formation of mature erythrocytes in the control cultures (day 21). In the beta-KD cultures, rare enucleated cells with pale blue cytoplasm were identified as well as occasional hemoglobinized cells (Figure 2H).

### Effects of Beta-KD Knockdown on the Erythroblast Growth and Differentiation

Cell counts performed on culture days 14 and 21 from three independent donors demonstrated a significant reduction in proliferation during the second phase of culture. Average cell counts of beta-KD on culture day 14 showed a significant 3.3 fold reduction compared to control (control = 3.4×10^5^±7.9×10^4^ cells/ml vs. beta-KD = 1.0×10^5^±1.9×10^4^ cells/ml). On culture day 21, the average cell counts were further increased in control cell cultures, but remained unchanged in the beta-KD cells (control = 5.6×10^5^±8.1×10^4^ cells/ml vs. beta-KD = 1.1×10^5^±2.6×10^4^ cells/ml).

**Figure 3:**
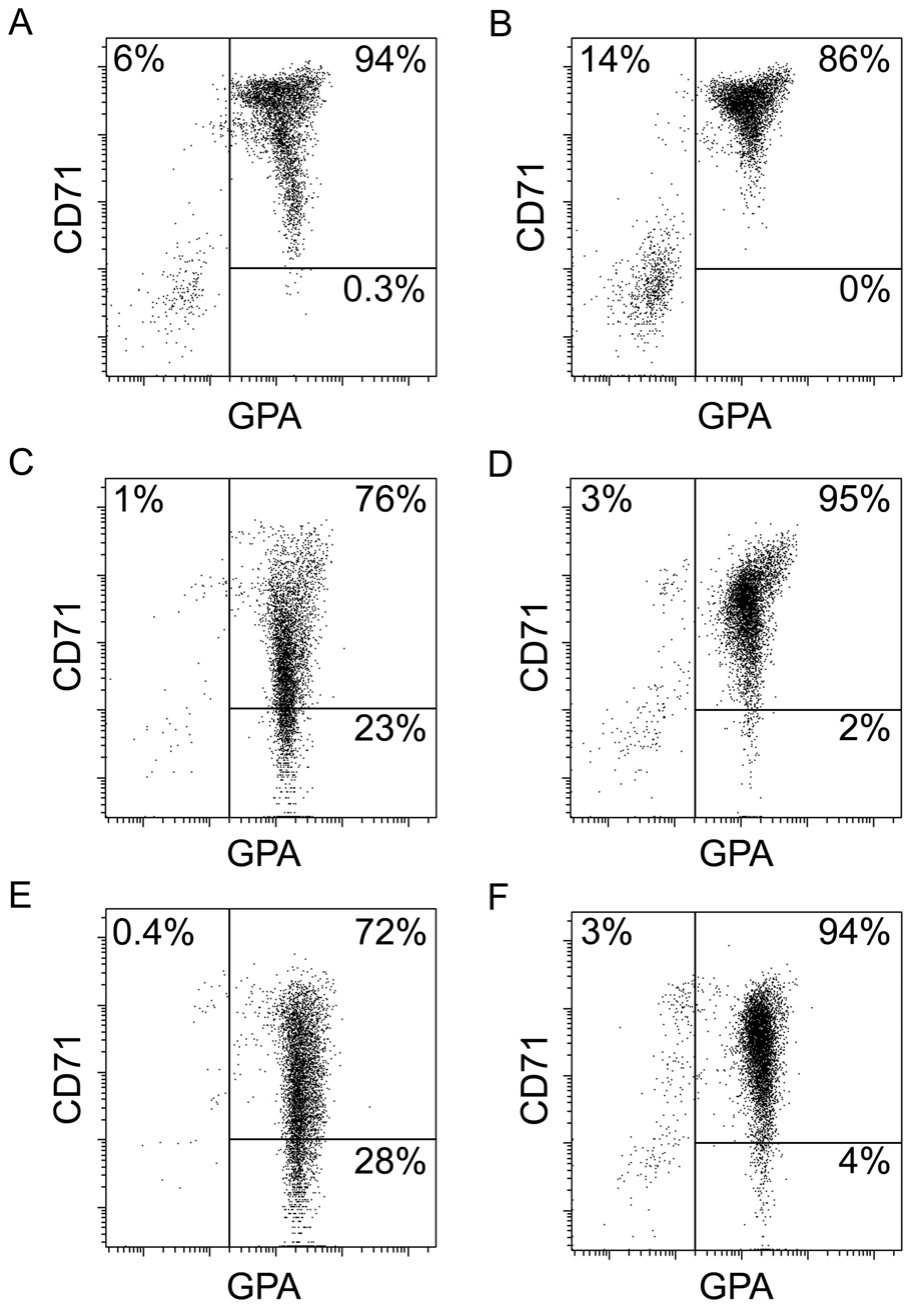
Flow cytometry analysis of terminal differentiation. Representative dot plots from (A) culture day 14 control erythroblasts, (B) culture day 14 beta-KD erythroblasts, (C) culture day 18 control erythroblasts, (D) culture day 18 beta-KD erythroblasts, (E) culture day 21 control erythroblasts, and (F) culture day 21 beta-KD erythroblasts. Cells were double stained with glycophorin A (GPA) and transferrin receptor (CD71).

Cell maturation was defined by expression of erythroid markers CD71 and GPA as previously described [9]. Representative data are shown in Figure 3 with descriptive statistics from triplicate experiments provided in Table S1. The main population on culture day 14 consisted of CD71 high/GPA(+) cells (proerythroblast stage of differentiation) in both control and beta-KD, respectively. As the cells undergo the final stages of differentiation, there is a subsequent loss of CD71. In the beta-KD cells, a significantly lower percent of GPA(+)/CD71(-) cells was detected compared to control in culture day 18 cells (representative data shown in Figures 3C, 3D; triplicate experiments: GPA(+)/CD71(-); control = 28.1±5.8% vs. beta-KD = 1.6±0.5%, *p* = 0.02). On culture day 21, the cellular phenotypes were similar to those on culture day 18 suggesting the absence of further differentiation (representative data shown in Figures 3E, 3F; triplicate experiments: GPA(+)/CD71(-); control = 17.6±6.3% vs. beta-KD = 3.6±2.5%, *p* = 0.03). These results suggest globin chain imbalances affect both the proliferative potential and differentiation of the beta-KD cells.

### Western Analysis of Soluble and Membrane Insoluble Globin Fractions

**Figure 4:**
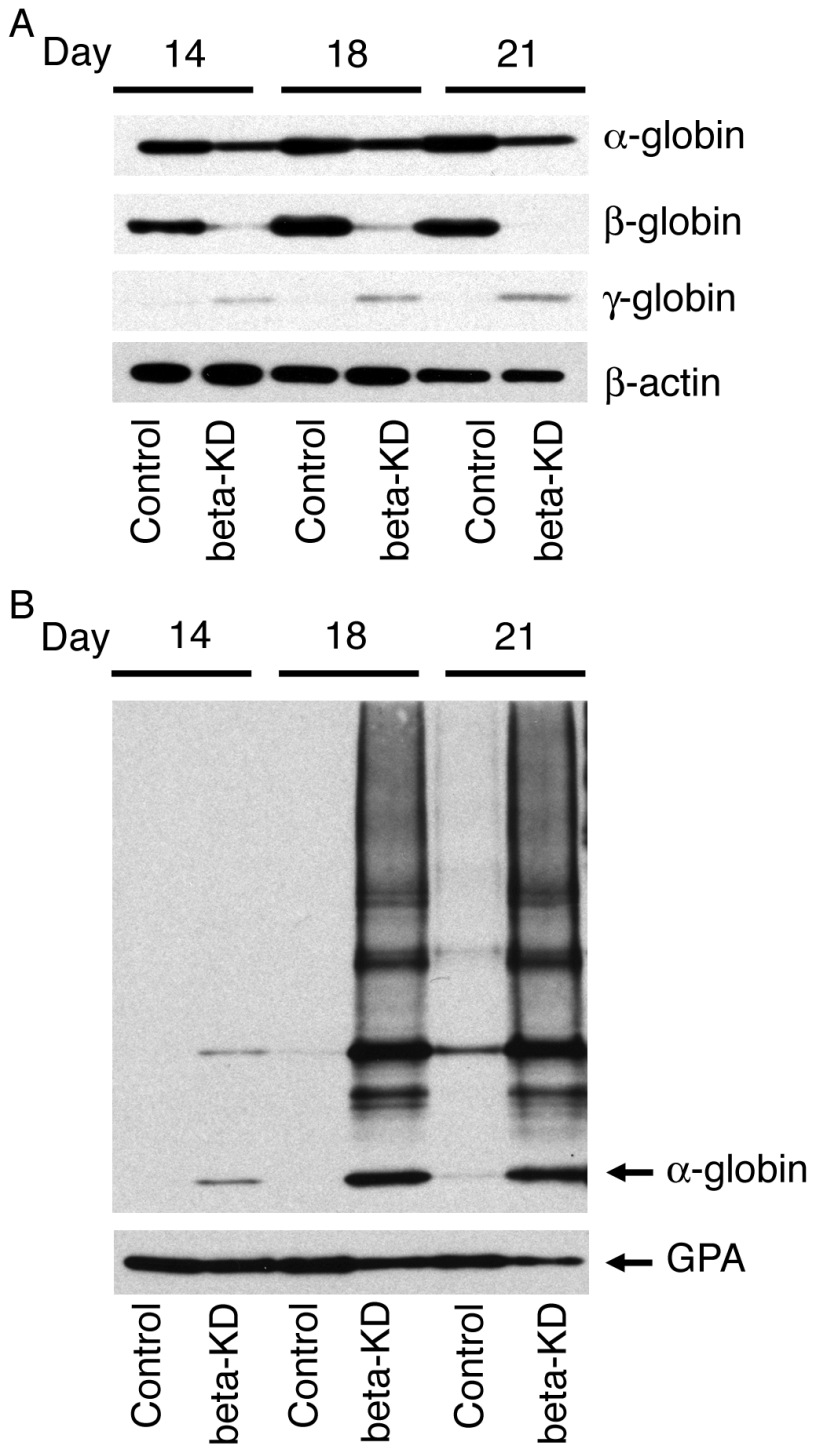
Soluble and insoluble globin analyses. (A) Western blot analyses of globin chains were performed using total soluble cytosolic protein (20 µg/lane) from day 14, 18, and 21 cultured erythroblasts. Antibodies against alpha-globin, beta-globin, and gamma-globin were used for comparison. Beta-actin was used as a loading control. (B) Western analysis of the alpha-globin insoluble membrane fraction on culture days 14, 18, and 21 of control and beta-KD cultured erythroblasts. Fractions from equivalent cell number preparations were used in each lane. GPA was used as a loading control for the insoluble membrane proteins. Equivalent film exposure time (one minute) was used for alpha-globin, beta-globin, and gamma-globin membrane comparisons.

Western analyses were performed to demonstrate the effects of beta-globin chain imbalance upon alpha-, beta- and gamma-globin protein expression during terminal differentiation. Representative results are shown in Figure 4A of three separate donors. These results are consistent with reduced beta-globin gene expression, and beta-globin protein was also significantly reduced. Statisitcal analyses of Western blot band intensities from three independent donors were compared for all globins and normalized to the loading control (beta-actin) on culture days 14, 18 and 21 (Table S2). The levels of cytosolic alpha-globin were significantly lower in the beta-KD cells; however, the level of reduction was less robust than that of beta-globin. Although gamma-globin was increased in the beta-KD samples, the increases did not reach statistical significance (Table S2).

Since human alpha-globin chains do not assemble into soluble hemoglobin species, the globin chain imbalance caused by beta-thalassemia results in an excess of free alpha-globin chains. The excess alpha-globin chains lose their solubility and precipitate in the insoluble membrane fraction of erythrocytes and erythroblast precursor cells as a hallmark of the disease [15]. Those precipitates cause oxidative damage and contribute to the cellular demise. To investigate whether the decreases in soluble alpha- globin chains on culture days 14, 18 and 21 were accompanied by increased insoluble alpha-globin in the beta-KD cells, we studied the insoluble membrane fractions [13] from culture day 14, 18 and 21 erythroblasts (Figure 4B). On culture day 14, alpha-globin was barely detectable in the insoluble beta-KD extracts. In extracts from more mature erythroblasts on culture day 18, major accumulation of insoluble alpha-globin was detected compared with the controls. A smeared pattern of alpha-globin protein at higher molecular weights was seen. No further increase in insoluble alpha-globin occurred after culture day 18.

### Assessment of Apoptosis in Beta-KD Cells using Flow Cytometry

**Figure 5:**
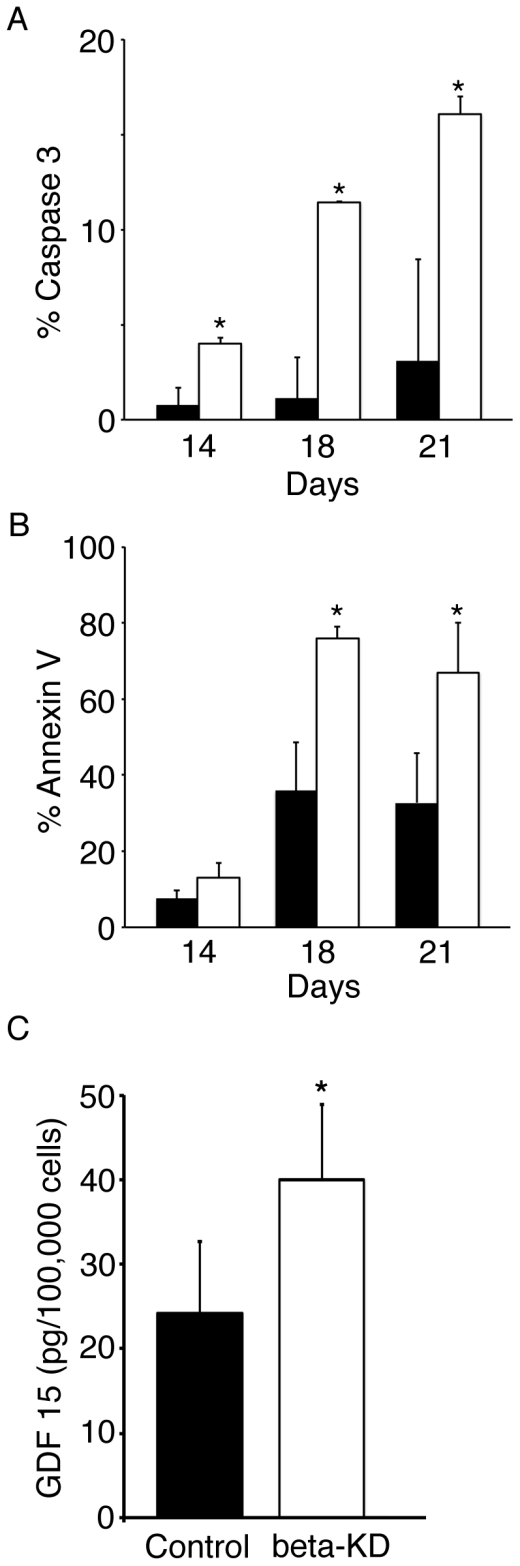
Analysis for markers of apoptosis. Erythroblasts collected on culture day 14, 18 and 21 were assessed for (A) early apoptosis marker active caspase-3 and (B) late apoptosis marker Annexin V. (C) GDF15 protein level in culture day 21 supernatants. Each panel shows average values from three separate donors, control (black bar) and beta-KD (open bar). Standard deviation bars are shown, and asterisks signify statistical significance of *p*<0.05.

Insoluble alpha-globin precipitation causes oxidative damage and apoptosis of erythroblasts in humans with beta-thalassemia [16], [17]. Assessment of caspase-3 [18] and Annexin V [19] expression were explored to determine if beta-KD similarly caused apoptosis *ex vivo*. On culture day 14, there was a small but significant increase in active caspase-3 that was detected in the beta-KD cells compared to controls (beta-KD = 4.0±1.0% vs. control = 0.7±0.3%, *p* = 0.02) suggesting that apoptosis is initiated relatively early during erythroblast maturation (Figure 5A). There was an additional increase in active caspase-3 from culture day 14 to 18 in beta-KD erythroblasts. Conversely, Annexin V staining was slightly increased, but did not achieve statistical significance on culture day 14. However, by culture day 18, when orthochromatic normoblasts are the prevalent population in control cultures, Annexin staining indicated that the majority of the population was comprised of apoptotic cells in beta-KD (beta-KD = 75.8±3.3%, vs. control = 35.9±12.7%, *p* = 0.02) (Figure 5B). As such, the data demonstrate early signs of apoptosis on culture day 14 followed by a significant increase later in the culture period, which coincides with the accumulation of insoluble alpha-globin in the cells. GDF15, a marker of erythroblast apoptosis that is usually increased in the serum of patients with thalassemia, was also increased in the culture supernatants of the beta-KD cells (Figure 5C). Increased apoptosis during the later stages of beta-KD differentiation, as well as a significant increase in GDF15 expression represent characteristic features of ineffective erythropoiesis identified in human beta-thalassemia [5], [14].

## Discussion

Here we report an artificially engineered model of thalassemia for *ex vivo* studies of human erythropoiesis developed using a shRNA lentiviral vector to reduce beta-globin expression in primary human erythroblasts. With this approach, terminal differentiation from proerythroblasts to orthochromatic normoblasts and enucleated cells occurs from culture days 14–18 (Figure 3). Cultures were maintained for an additional three days (days 18–21) to explore the potential for further differentiation of the beta-KD cells. Overall, the strategy of knocking down beta-globin mRNA and protein expression resulted in phenotypic changes consistent with those predicted for severe beta-thalassemia in humans. The efficiency of the tested shRNA clone produced a greater than 90% reduction in the levels of cellular beta-globin mRNA when compared to control cells. Since the shRNA clone (TRCN0000232626) also targets delta-globin mRNA [20], delta-globin mRNA levels were reduced as shown by QPCR (Figure 1A). However, gamma-globin levels increased slightly as determined by QPCR (Figure 1A) and Western analyses (Figure 4A). By comparison, alpha-globin mRNA remained stable. Major reductions in adult hemoglobin (HbA) and beta-globin chains were also detected at the protein level. By the end of the culture period, fetal hemoglobin (HbF) was the dominant hemoglobin variant in beta-KD cells mainly because of the massive reduction in HbA. The increase in HbF among the mature beta-KD cells may have also caused improved survival of some cells during differentiation [21]. The terminal stages of differentiation in beta-KD cells were marked by apoptosis of most cells, and only a thin ring of hemoglobinized cytoplasm in many of the remaining orthochromatic normoblasts. The high levels of detected apoptosis reflect the magnitude of beta-globin reduction, as well as the absence of macrophage clearance of the apoptotic cells by phagocytosis.

Balanced globin chain synthesis coupled with heme biosynthesis is required to produce sufficient quantities of hemoglobin for effective erythropoiesis [22]. In beta-thalassemia major, the severe chain imbalance of excess alpha-globin chains leads to the formation of hemichromes (alpha-globin/heme aggregates), which cause precipitates in the erythrocyte membrane [15]. Those precipitates produce ROS damage and cell death. In beta-KD cultures, soluble alpha-globin protein was reduced during the final stages of maturation in association with deposition of insoluble alpha-globin in the membranes. The cause for the smeared pattern of insoluble alpha hemoglobin seen in the Western analyses is unknown, but may be related to the absence of macrophage clearance of the apoptotic cells or ubiquitination of the free alpha-globin chains [13]. The accumulation of free alpha-globin in the membranes coincided with apoptosis of the beta-KD cells during the period of maturation when hemoglobin accumulates in donor-matched control cells.

Despite several similarities between the *ex vivo* beta-KD model and clinical defects in erythropoiesis reported *in vivo* for beta-thalassemia, it must be emphasized that the findings do not completely reflect the beta-thalassemia phenotype. The experimental design did not include mixed cell cultures, so potential roles for macrophages in the production or clearance of viable or apoptotic erythrocytes were not explored. Since the beta-KD model reflects erythroblast differentiation in the absence of additional cell membrane alterations that occur *in vivo,* microcytosis was not evident [23], [24]. Among the enucleated cells, hypochromia was obvious as several cells demonstrated an erythroid ghost-like appearance. However, the low number of enucleated beta-KD cells prevented more formal analyses of those cells. Enucleated cells were reportedly absent from *ex vivo* cultures of erythroblasts obtained from blood of thalassemia patients [25]. While we were unable to obtain the appropriate clinical samples for direct comparisons to be made between *ex vivo* cultures of beta-KD versus thalassemia-major erythroblasts as part of this study, such comparisons should be made in laboratories where those cells are available.

As demonstrated from other areas of biomedical research [26], there exists an urgent need for disease models using primary human cells or tissues. Considerable resources have been utilized in recent decades to develop and explore murine models of hemoglobinopathies. Despite their robustness for understanding murine erythropoiesis *in vivo*, those disease models have not yet led to successful clinical trials for improving the health for patients with thalassemia. Differences between the kinetics of murine and human erythroblast differentiation [27], iron biology [7], [28]–[30], and globin gene regulation [31] confound our ability to interpret the murine data in the context of clinical application. As a result, a considerable gap is developing between basic and clinical research for beta-thalassemia.

This study was undertaken to characterize the effects of beta-globin chain imbalances upon *ex vivo* erythroblast differentiation and survival using primary human cells. Our model for knockdown of beta-globin gene expression may also permit examinations of dysfunctional erythroblast heme metabolism or mitochondrial function caused by globin chain imbalance [32]. Investigations of erythroblast GDF15 regulation [14] or caspase related GATA-1 expression [33] can be explored further. Based upon its simplicity and reproducibility, the model is being developed for preclinical assessment and comparison with beta thalassemia-related discoveries made in other model systems including biochemical assays, immortalized cell lines, or genetically modified rodents [22]. Since the shRNA in this study does not target gamma-globin mRNA, it should be possible to screen and study fetal hemoglobin augmenting drugs or small molecules for their potential to correct the beta-thalassemia phenotype.

## Supporting Information

Table S1 (DOCX)

Table S2 (DOCX)
