# Supplementary material for: A Synthetic Model of Human Beta-Thalassemia Erythropoiesis Using CD34+ Cells from Healthy Adult Donors
Source: PLoS One. 2013 Jul 8;8(7):e68307. doi: 10.1371/journal.pone.0068307 (PMC3704632; doi:10.1371/journal.pone.0068307)
Supplement: Table S1 — (DOCX) [file pone.0068307.s001.docx]

|  |  | Control | Beta-KD | *t-*test |
| --- | --- | --- | --- | --- |
| Day 14 | GPA(-) | 7.5+5.6 | 11.8+1.8 | 0.363 |
|  | GPA(+)/CD71(+) | 92.0+5.2 | 88.2+1.8 | 0.380 |
|  | GPA(+)/CD71(-) | 0.5+0.5 | 0 | 0.250 |
| Day 18 | GPA(-) | 1.6+1.3 | 3.3+0.7 | 0.051 |
|  | GPA(+)/CD71(+) | 70.4+7.1 | 95.1+0.8 | 0.022* |
|  | GPA(+)/CD71(-) | 28.1+5.8 | 1.6+0.5 | 0.016* |
| Day 21 | GPA(-) | 0.7+0.2 | 2.2+1.0 | 0.162 |
|  | GPA(+)/CD71(+) | 81.5+6.2 | 94.3+2.3 | 0.030* |
|  | GPA(+)/CD71(-) | 17.6+6.3 | 3.6+2.5 | 0.034* |

**Table S1**

**Table S1 :**

Statistical analyses of flow cytometry dot plots comparing percentages of cells according to Glycophorin A (GPA) and Transferrin Receptor (CD71) expression patterns. Percentages from control and beta-KD cellular populations on culture days 14, 18 and 21 represent mean values with standard deviation from triplicate experiments. Also see corresponding Figure 3A-3F. Asterisks signify statistical significance of *p*<0.05.
