# Supplementary material for: A Synthetic Model of Human Beta-Thalassemia Erythropoiesis Using CD34+ Cells from Healthy Adult Donors
Source: PLoS One. 2013 Jul 8;8(7):e68307. doi: 10.1371/journal.pone.0068307 (PMC3704632; doi:10.1371/journal.pone.0068307)
Supplement: Table S2 — (DOCX) [file pone.0068307.s002.docx]

**Table S2**

|  |  | Control | Beta-KD | *t-*test |
| --- | --- | --- | --- | --- |
| Alpha-globin | Day 14 | 1 | 0.76+0.06 | 0.023* |
|  | Day 18 | 1 | 0.75+0.15 | 0.010* |
|  | Day 21 | 1 | 0.74+0.10 | 0.046* |
| Beta-globin | Day 14 | 1 | 0.02+0.03 | 0.000* |
|  | Day 18 | 1 | 0.08+0.06 | 0.002* |
|  | Day 21 | 1 | 0.08+0.08 | 0.002* |
| Gamma-globin | Day 14 | 1 | 2.47+0.84 | 0.095 |
|  | Day 18 | 1 | 3.21+2.70 | 0.293 |
|  | Day 21 | 1 | 3.94+3.40 | 0.272 |

**Table S2 :**

Statistical analyses of the band intensities for soluble globin proteins, normalized to β-actin performed in triplicate experiments corresponding to Figure 4A. Band intensities from control and beta-KD Western analyses were compared on culture days 14, 18 and 21. Beta-KD represent mean values relative to the control with standard deviation from triplicate experiments (actin normalized value for each control sample was defined as 1). Asterisks signify statistical significance of *p*<0.05.
